# 1,4-dihydroxy-2-naphthoic Acid Induces Apoptosis in Human Keratinocyte: Potential Application for Psoriasis Treatment

**DOI:** 10.1155/2013/792840

**Published:** 2013-04-11

**Authors:** Chong-Fai Mok, Chuan-Ming Xie, Kathy Wai-Yan Sham, Zhi-Xiu Lin, Christopher Hon-Ki Cheng

**Affiliations:** ^1^School of Biomedical Sciences, Faculty of Medicine, The Chinese University of Hong Kong, Shatin, N.T., Hong Kong, China; ^2^School of Chinese Medicine, Faculty of Science, The Chinese University of Hong Kong, Shatin, N.T., Hong Kong, China; ^3^Centre of Novel Functional Molecules, The Chinese University of Hong Kong, Shatin, N.T., Hong Kong, China

## Abstract

Psoriasis, which affects approximately 1–3% of the population worldwide, is a chronic inflammatory skin disorder characterized by epidermal keratinocytes hyperproliferation, abnormal differentiation, and inflammatory infiltration. Decrease in keratinocyte apoptosis is a specific pathogenic phenomenon in psoriasis. Chinese herbs have been used for the treatment of psoriasis in China showing promising effect in clinical trials. A traditional Chinese medicine has relatively fewer side effects with longer remission time and lower recurrence rate. The extract of *Rubia cordifolia* L. (EA) was previously found by us to induce HaCaT keratinocytes apoptosis. In this study we identified one of the components in *Rubia cordifolia* L., the anthraquinone precursor 1,4-dihydroxy-2-naphthoic acid (DHNA), induces HaCaT keratinocytes apoptosis through G0/G1 cell cycle arrest. We have also demonstrated that DHNA acts through both caspase-dependent and caspase-independent pathways. Besides, cytotoxicity and IL-1**α** release assays indicate that DHNA causes less irritation problems than dithranol, which is commonly employed to treat psoriasis in many countries. Since DHNA possesses similar apoptotic effects on keratinocytes as dithranol but causes less irritation, DHNA therefore constitutes a promising alternative agent for treating psoriasis. Our studies also provide an insight on the potential of using EA and DHNA, alternatively, as a safe and effective treatment modality for psoriasis.

## 1. Introduction

Psoriasis, affecting 1–3% of the population worldwide [1], is a common chronic immune disorder characterized by thickened red plaques with an overlying silver-white scale. The most common type of psoriasis is psoriasis vulgaris which accounts for 90% of the cases [2]. Affecting both sexes equally and people of all ages; psoriasis causes significant impacts on the quality of life [3]. Conventional treatments of psoriasis such as topical, phototherapy, and systemic treatment are based on severity of disease. Since an estimated 75% of psoriatic patients have mild-to-moderate disease, topical treatments remain the most widely used [1]. The costs for treating patients with psoriasis are substantial, estimated to be US $1.6–3.2 billion/year and $1,130 to 6,650 per patient/year [4, 5]. With recent introduction of biological agents, the estimated cost can range from US $13000 to 30000/patient annually [6].

Psoriasis has three principal histological features, including epidermal hyperplasia or abnormal differentiation, dilated blood vessels in dermis, and predominantly infiltration of leucocytes into dermis causing inflammation. It is considered a T-helper 1 (Th1) disease based on the increase in cytokines of the Th1 pathway such as interferon gamma, interleukin (IL) 2 and 12, as found in psoriatic plaques [2, 3]. However, a strong evidence also indicated keratinocytes contributed to the disease [7] and keratinocytes is viewed as another major player of this chronic inflammatory disease [8]. For instance, decrease in skin cells apoptosis is suggested as a specific pathogenic phenomenon in psoriasis [9, 10]. Acanthosis in psoriatic skin is resulted from diminished epidermal apoptosis [11], while induction of apoptosis is involved in the regression of psoriatic hyperplasia [9, 10]. Therapies based on the regulation of keratinocyte proliferation are potentially useful in treatment of psoriasis because the restored homeostatic control of keratinocyte growth and differentiation is crucial for recovery from psoriatic to normal epidermis [12]. For example, established therapies like dithranol, vitamin-D_3_ analogs, and low-dose methotrexate induce apoptosis [11].

Chinese herbs have been used for the treatment of psoriasis in China, and clinical trials have also been carrying out [13, 14] with promising evidence of effectiveness [15, 16]. In traditional Chinese medicine (TCM) theories, psoriasis is classified into three categories: blood heat, blood dryness, and blood stasis [13]. The rationale for using TCM is related to their actions on clearing internal heat, activating blood to remove stasis, and strengthening any deficiencies. The literature review also describes the mechanism of the TCM treatment in psoriasis is through inhibition of keratinocyte proliferation and induction of apoptosis [17]. Besides, therapeutic actions of these herbs include anti-inflammatory, modulation of cytokine production, or inhibition of angiogenesis which are relevant in reducing the severity of psoriasis [13, 17]. Although Western medicine is practiced primarily for the treatment of psoriasis, patients' dissatisfaction with conventional treatment or adverse effects after application were the most common reasons for switching to alternative therapies like herbal remedies. Besides, reviews also reported that TCM has relatively fewer side effects, a longer remission time, and a lower recurrence rate than the Western therapeutic agents [18]. Consistent with this finding, one study showed that the recurrence rate of psoriasis in TCM treated group was significantly lower than Daivonex (calcipotriol) treated group while the clinical efficacy was similar [19].

**Figure 1:**
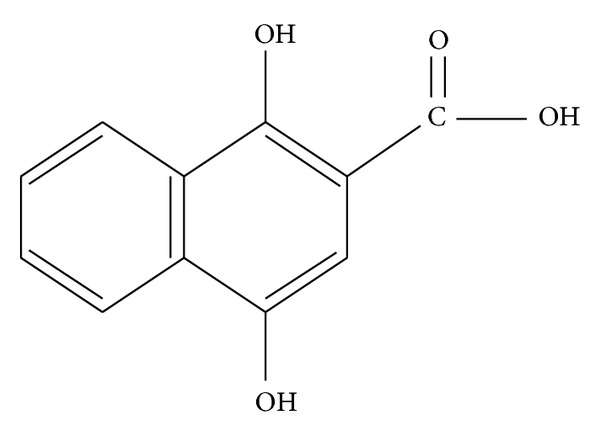
The structure of 1,4-dihydroxy-2-naphthoic acid (DHNA).

In our previous studies it was demonstrated that the extract of *Rubia cordifolia *L. could induce keratinocyte apoptosis in a psoriasis-relevant HaCaT cell models [12, 20, 21]. In this study one of the components in *Rubia cordifolia *L., the anthraquinone precursor DHNA (Figure 1), was found to induce HaCaT keratinocytes apoptosis through G0/G1 cell-cycle arrest, activation of caspases as well as in a caspase independent manner. Moreover, results from cytotoxicity and IL-1*α* release assay suggest DHNA causes less irritation problems than dithranol, which its use has declined because of its substantial skin irritation problems, but is still a popular and important topical treatment for psoriasis among European countries. Since DHNA possesses similar apoptotic effects on keratinocytes as dithranol but causes less irritation, these results suggest DHNA may be developed into a less-irritating topical agent alternative to dithranol for the treatment of psoriasis. In addition, the ethyl acetate extract of the root of *Rubia cordifolia *L. and DHNA may be used together in a sequential therapy as a safe and effective therapeutic alternative for the treatment of psoriasis.

## 2. Materials and Methods

### 2.1. Reagents

Antibodies for Bcl-2, Bcl-xL, p21, p27, Cyclin D1, Cdk2, *β*-actin were purchased from Santa Cruz Biotechnology (Santa Cruz, CA, USA); antibodies for caspase-3/7/8/9, PARP, Bid, Fas, FADD, Cyclin A/D3, Cdk4/6, AIF and endoG were from Cell Signaling Technologies (Beverly, MA, USA). All other reagents were from Sigma-Aldrich (St. Louis, MO, USA). DMEM, RPMI 1640 medium and fetal bovine serum (FBS) were purchased from Gibco Laboratories (NY, USA). Medium 254 with Human Melanocyte Growth Supplement-2 was purchased from Cascade Biologics/Invitrogen (Portland, OR, USA).

### 2.2. Cell Culture

The spontaneously immortalized human epidermal keratinocyte HaCaT cells were purchased from China Centre for Type Culture Collection, Wuhan, China. The human foreskin fibroblast Hs-68 cells were purchased from the American Type Culture Collection (Manassas, VA, USA). The human undifferentiated keratinocyte NCTC 2544 cells were provided by Interlab Cell Line Collection (Genoa, Italy) and the immortalized human epidermal melanocyte PIG1 cells were a gift from Dr Caroline Le Poole (Loyola University Chicago, Maywood, IL, USA). HaCaT and Hs-68 cells were maintained in DMEM, NCTC 2544 in RPMI 1640 and PIG1 cells in Medium 254 with Human Melanocyte Growth Supplement-2, respectively. All cells were cultured in complete medium containing 10% FBS, 100 *μ*g/mL of streptomycin, and 100 U/mL of penicillin (Gibco Laboratories) at 37°C in a humidified atmosphere of 5% CO_2_. Cell culture experiments were carried out at 60–80% confluent.

### 2.3. Proliferation Assay

The ethyl acetate extract (EA) of *Rubia cordifolia *L. was isolated as described [21]. 1,4-dihydroxy-2-naphthoic acid (98%), dithranol (≥90%), and EA were dissolved in DMSO as stock. All cells were seeded and treated with various concentrations of DHNA, dithranol or EA. After treatment, cells were fixed with cold 50% trichloroacetic acid and incubated 1 h at 4°C. Cell was then washed with deionized water, air-dried, and stained with 0.4% (w/v) sulphorhodamine B for 30 min at room temperature. Unincorporated dye was removed with 1% acetic acid wash and the cell was again air-dried. Incorporated dye was then solubilized in 10 mM Tris base and absorbance was measured at 565/690 nm using a Sunrise microplate spectrophotometer (Tecan, Switzerland). IC_50_ were determined using GraphPad Prism 5.0 (GraphPad Software, San Diego, CA, USA).

### 2.4. Fluorescent Staining for Morphological Evaluation

After DHNA treatment, HaCaT cells were fixed in 4% paraformaldehyde for 30 min, washed with phosphate-buffered saline (PBS) and then stained with 2 *μ*g/mL Hoechst 33342 (Molecular Probes, Eugene, OR, USA) for 15 min at room temperature in the dark. Morphology was evaluated using an Axiophot fluorescence microscope (Carl Zeiss, Oberkochen, Germany).

### 2.5. Annexin V/Propidium Iodide Staining

After DHNA treatment, HaCaT cells were resuspended in annexin V binding buffer (10 mM HEPES, 140 mM NaCl, and 2.5 mM CaCl_2_, pH 7.4) and then incubated with FITC-conjugated annexin V (Biosource International, Inc., USA), and 0.5 *μ*g/mL propidium iodide (PI) for 15 min at room temperature in the dark. Cells were analyzed on a FACSCanto flow cytometer (BD Biosciences, San Jose, CA, USA) immediately and then analyzed using WinMDI 2.9 (developed by Dr. J. Trotter, Scripps Institute, La Jolla, CA, USA). The total number of early (FITC+/PI−) and late (FITC+/PI+) apoptotic cells was assessed. In another experiment, HaCaT cells were preincubated with 40 *μ*M pan-caspase inhibitor Z-VAD-FMK (BD Pharmingen, San Diego, CA, USA) for 1 h before exposure to DHNA.

### 2.6. JC-1 Staining

After DHNA treatment, HaCaT cells were harvested and washed with PBS. Cells were then incubated in 1.5 *μ*M JC-1 (5,5′,6,6′-tetrachloro-1,1′,3,3′-tetraethylbenzimidazolylcarbocyanine iodide, Molecular Probes) in PBS at 37°C for 15 min in the dark. Thereafter cells were washed, resuspended in PBS and then immediately analyzed on the FACSCanto flow cytometer. If mitochondrial membrane potential (MMP) depolarizes, JC-1 becomes a monomer and emits green fluorescence; when MMP polarizes, JC-1 becomes a polymer and emits red fluorescence. It indicates mitochondria depolarization by a decrease in red/green fluorescence intensity ratio.

### 2.7. Cell Cycle Analysis

HaCaT cells were treated with DHNA for 24 h, fixed with 75% ice-cold ethanol at 4°C for 1 h and stained with PI/RNase solution (BD Pharmingen) for 15 min at room temperature in the dark. Stained cells were analyzed using the FACSCanto flow cytometer immediately. Cell cycle distribution was calculated with ModFit LT 3.1 (Verity Software House, Topsham, ME, USA).

### 2.8. Detection of DNA Fragmentation

After incubation of HaCaT cells with DHNA, subsequent experimental steps were following the protocol of the Cell Death Detection ELISA^plus^ (Roche Applied Science, Basel, Switzerland). Absorbance was measured at 405/490 nm by the Sunrise microplate spectrophotometer. Enrichment of mono- and oligonucleosomes (cytoplasmic histone-associated DNA fragments) into the cytoplasm was calculated as absorbance of treatment cells/absorbance of control (enrichment factor) which was used as a parameter of apoptosis.

### 2.9. Terminal Deoxynucleotidyltransferase-Mediated dUTP Nick End Labeling (TUNEL) Assay

After DHNA treatment, HaCaT cells were collected and washed and subsequent steps were following the protocol of *In Situ* Cell Death Detection kit, Fluorescein (Roche Applied Science). Fluorescein-labeled DNA strand breaks were detected by flow cytometry. The percentage of hypodiploid (fragmented) nuclei reflecting relative proportion of apoptotic cells was analyzed by WinMDI 2.9.

### 2.10. Western Blot Analysis

After DHNA treatment HaCaT cells were collected and lysed in CelLytic M Mammalian Cell Lysis/Extraction Reagent with freshly added Protease Inhibitor Cocktail for 30 min at 4°C. Cell lysate was centrifuged at 15000 g for 15 min at 4°C and supernatant was collected as whole cell lysate. Protein concentration was determined by Bio-Rad protein assay dye reagent concentrate (Bio-Rad Laboratories, Hercules, CA, USA) following the manufacturer's instructions. Whole cell lysate was denatured by boiling for 5 min in 6X SDS loading buffer (0.375 M Tris [pH 6.8], 30% glycerol, 12% SDS, 0.2% bromophenol blue and 12%  *β*-mercaptoethanol). Equal amount of protein (10–20 *μ*g) was then separated by 12–15% SDS-PAGE and later proteins were transferred to polyvinylidene difluoride membrane (Millipore, Billerica, MA, USA) using Trans-Blot SD semidry transfer cell (Bio-Rad Laboratories). After that, membranes were blocked with 2–5% (w/v) bovine serum albumin or nonfat dry milk in Tris-buffered Saline-Tween (TBS-T; 0.05% v/v Tween-20, 10 mM Tris [pH 7.4], 150 mM NaCl) for 1 h and subsequently incubated overnight at 4°C with different primary antibodies in TBS-T. Membrane was then washed using TBS-T and followed by incubation with corresponding horseradish peroxidase labeled second antibodies (Santa Cruz Biotechnology) in TBS-T for 1 h. After washing of membrane with TBS-T, protein bands were visualized using Immobilon Western Chemiluminescent HRP Substrate (Millipore) and autoradiography with Lumi-Film Chemiluminescent Detection Film (Roche Applied Science). Equal protein loading was monitored by *β*-actin antibody on the same membrane.

### 2.11. Immunofluorescence Staining

HaCaT cells were first seeded on cover-slipped 6 well plates. After DHNA treatment, cells were washed three times with PBS and fixed in 1 : 1 acetone/methanol at −20°C for 10 min. Thereafter, cells were washed with PBS three times and then blocked for 15 min in blocking solution (1% BSA in PBS) at room temperature. After that, cells were incubated with either one of the following antibodies in blocking solution overnight at 4°C: apoptosis inducing factor, AIF or endonuclease G, and endoG. Cells were then washed with PBS three times and incubated with corresponding secondary antibody conjugated to Alexa Fluor 488 (Molecular Probes) in blocking solution in dark at room temperature for 1 h. After further washing with PBS for three times, the cover slips were then mounted on glass slides using the Vectashield mounting medium with DAPI (Vector Laboratories, Burlingame, CA). Stained cells were then visualized under the Nikon Ti-E inverted microscope (Nikon, Tokyo, Japan).

### 2.12. IL-1*α* Release Assay

NCTC 2544 cells were seeded and treated with various concentrations of DHNA or dithranol for 72 h. After incubation, the microplate was centrifuged and conditioned medium was collected to determine the extracellular IL-1*α* (IL-1*α* release) using an IL-1*α* ELISA kit (Diaclone Research, France). All other steps were then according to the manufacturer's protocol. Results were expressed in pg/mL.

### 2.13. Statistical Analysis

Data were expressed as mean ± SEM. Statistical comparisons between DHNA treatment and control were carried out using one-way analysis of variance (ANOVA), followed by post hoc Dunnett's test using GraphPad Prism 5.0 (GraphPad Software, San Diego, CA, USA). Differences were considered significant at *P* < 0.05.

## 3. Results

### 3.1. DHNA Inhibits Proliferation of Human Epidermal HaCaT Keratinocyte

DHNA, dithranol and EA inhibited proliferation of partially differentiated and undifferentiated human keratinocyte (HaCaT and NCTC 2544) in time and concentration-dependent manners (Table 1). Compared with dithranol and, EA, DHNA exhibited weaker growth inhibition on both keratinocyte at 48 and 72 h. On the other hand, DHNA exerted lower cytotoxic effect on Hs-68 fibroblast (226.3 *μ*M or 46.21 *μ*g/mL) and is comparable with EA (43.95 *μ*g/mL), while dithranol had a more potent cytotoxic effect (27.42 *μ*M or 6.20 *μ*g/mL). PIG1 melanocyte is the most sensitive towards DHNA or dithranol; still DHNA (23.98 *μ*M or 4.90 *μ*g/mL) is less cytotoxic than dithranol (2.34 *μ*M or 0.53 *μ*g/mL).

### 3.2. Alteration of Cellular Morphology

**Figure 2:**
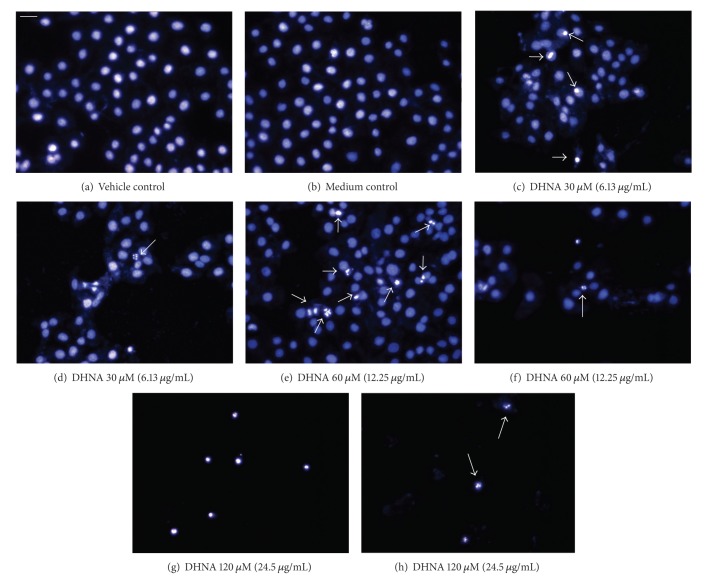
Action of DHNA on HaCaT cells morphology. (a) Vehicle (0.24% DMSO) and (b) medium only treated HaCaT cells. (c to h) HaCaT cells treated with 30 *μ*M (6.126 *μ*g/mL; (c, d)), 60 *μ*M (12.25 *μ*g/mL; (e, f)) and 120 *μ*M (24.5 *μ*g/mL; (g, h)) DHNA for 72 h. After staining with Hoechst 33342, morphological examinations were carried out using fluorescent microscope. Cells with chromatin condensation (c, e) and nuclear fragmentation (d, f, h) are indicated by arrows. In (g), all cells undergo chromatin condensation. Three independent experiments were performed with similar results. Scale bar 50 *μ*m.

Vehicle (≤0.24% DMSO) and medium incubated cells showed blue-fluorescent nuclei of normal nuclear morphology (Figures 2(a) and 2(b)). After exposure to 30 and 60 *μ*M (6.13 and 12.25 *μ*g/mL) DHNA for 72 h, a number of HaCaT cells increased in nuclear fluorescence from dark blue (control) to light blue/white, indicating chromatin condensation (Figures 2(c) and 2(e)). At 120 *μ*M (24.5 *μ*g/mL) DHNA, light blue/white fluorescence was emitted from the nuclei of all cells (Figure 2(g)). Besides, typical apoptotic phenomenon in term of nuclear fragmentation was also observed in all DHNA treated HaCaT cells (Figures 2(d), 2(f), and 2(h)).

### 3.3. DHNA Increases Phosphatidylserine Externalization in HaCaT Cells

**Figure 3:**
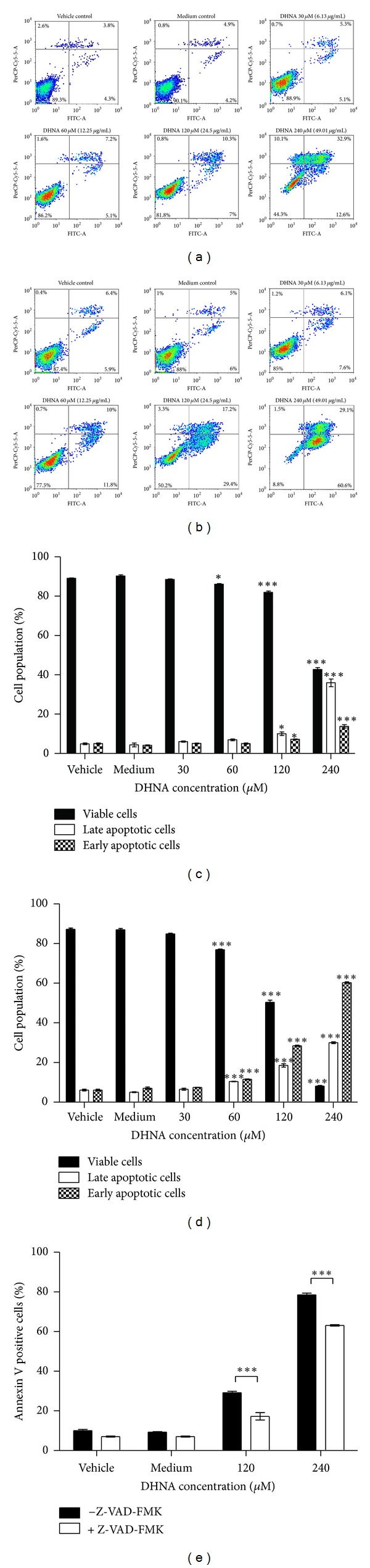
Phosphatidylserine externalization is increased in DHNA-treated HaCaT cells. (a, b) Density plots and (c, d) bar chart showing effects of the DHNA on distribution of viable (lower left) and early/late apoptotic (lower/upper right) HaCaT cells. HaCaT cells were treated with vehicle (0.24% DMSO), medium only or various concentration of DHNA for 9 (a, c) and 24 h (b, d) and then analyzed by Annexin V/PI and flow cytometry. (e) DHNA-induced apoptosis in HaCaT cells was reduced by pan-caspase inhibitor. HaCaT cells were pretreated with 40 *μ*M pan-caspase inhibitor Z-VAD-FMK for 1 h followed by DHNA treatment for 24 h. Three independent experiments with triplicates each time were performed with similar results. Data are expressed as mean ± SEM from one representative experiment and significant difference at *P < 0.05, ***P < 0.001 when versus vehicle control (c, d) or between DHNA treatment (e).

To observe the events of apoptosis, the effects of DHNA on HaCaT cells regarding the exposure of phosphatidylserine at the cell surface were observed at 9 h (Figures 3(a) and 3(c)) and 24 h (Figures 3(b) and 3(d)). Results were analyzed and presented as density plots (Figures 3(a) and 3(b)) and bar charts (Figures 3(c) and 3(d)). The distribution of viable, early apoptotic, and late apoptotic cells between vehicle and medium incubated HaCaT cell is similar (Figures 3(a)–3(d)). Compared with viable cells (FITC−/PI−) in the vehicle control (89.3% at 9 h, 87.4% at 24 h), the number of viable HaCaT cells after DHNA treatment decreased dramatically from 88.9% (30 *μ*M/6.13 *μ*g/mL; 9 h) to 44.3% (240 *μ*M/49.01 *μ*g/mL; 9 h) and from 85.0% (30 *μ*M/6.13 *μ*g/mL; 24 h) to 8.8% (240 *μ*M/49.01 *μ*g/mL; 24 h) (Figures 3(a) and 3(b)). At the same time, the percentages of total apoptotic fraction (early [FITC+/PI−] plus late [FITC+/PI+] apoptotic cells) after DHNA treatment also significantly increased from 10.4% (30 *μ*M/6.13 *μ*g/mL; 9 h) to 45.5% (240 *μ*M/49.01 *μ*g/mL; 9 h) and from 13.7% (30 *μ*M/6.13 *μ*g/mL; 24 h) to 89.7% (240 *μ*M/49.01 *μ*g/mL; 24 h) compared with vehicle control (8.1% at 9 h, 12.3% at 24 h) (Figures 3(a) and 3(b)). These findings indicated that DHNA induced apoptosis in HaCaT cells in time and dose dependent manners. In caspase inhibition assay, pretreatment of HaCaT cells with 40 *μ*M pan-caspase inhibitor Z-VAD-FMK partially blocked DHNA-induced apoptosis (Figure 3(e)).

### 3.4. DHNA Decreases MMP

**Figure 4:**
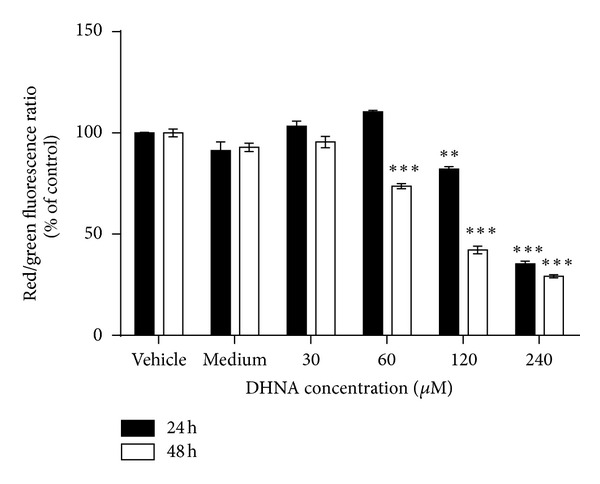
DHNA decreases MMP in HaCaT cells. HaCaT cells were treated with vehicle (0.24% DMSO), medium only, or various concentration of DHNA for 24 and 48 h. After staining with JC-1, HaCaT cells were analyzed by flow cytometry and the red/green fluorescence ratio in each sample was calculated. Three independent experiments with triplicates each time were performed with similar results. Data are expressed as mean ± SEM from one representative experiment and significant difference at **P < 0.01, ***P < 0.001 when versus vehicle control.

Apoptosis accompanies with the loss of MMP and it was measured by JC-1 staining in HaCaT cells after DHNA treatment. Compared with vehicle control, MMP in HaCaT cells decreased dramatically from 103.3% (30 *μ*M/6.13 *μ*g/mL; 24 h) to 35.4% (240 *μ*M/49.01 *μ*g/mL; 24 h) and from 95.5% (30 *μ*M/6.13 *μ*g/mL; 48 h) to 29.2% (240 *μ*M/49.01 *μ*g/mL; 48 h) after DHNA treatment, and the effect is time and dose-dependent (Figure 4).

### 3.5. DHNA Causes G0/G1 Cell Cycle Arrest in HaCaT Cells

**Figure 5:**
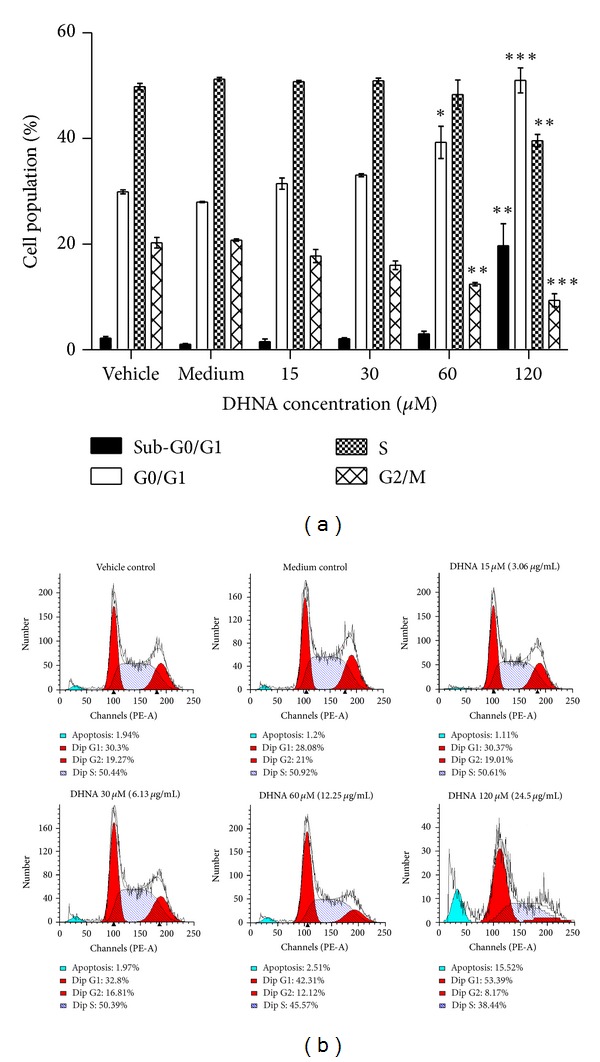
Effect of the DHNA on cell cycle distribution in HaCaT cells. (a) Bar chart and (b) graphical analysis showing the effect of the DHNA on cell cycle distribution of sub-G0/G1, G0/G1, S, and G2/M phase in HaCaT cells. HaCaT cells were treated with vehicle (0.24% DMSO), medium only or various concentration of DHNA for 24 h then stained with PI and cell cycle was analyzed by flow cytometry. The apoptotic proportion was recognized as the sub-G0/G1 population. Three independent experiments with triplicates each time were performed with similar results. Data are expressed as mean ± SEM from one representative experiment and significant difference at *P < 0.05, **P < 0.01, ***P < 0.001 when versus vehicle control.

The distribution of sub-G0/G1, G0/G1, S and G2/M phase cells between vehicle and medium treated HaCaT cell does not differ significantly (Figure 5). In HaCaT cells incubated with vehicle control, the percent distribution of sub-G0/G1, G0/G1, S, and G2/M phase cells are 2.2, 29.9, 49.8, and 20.3%, respectively, (Figure 5(a)). Treatment of DHNA dose-dependently induced G0/G1 cell cycle arrest in HaCaT cells at 24 h (Figure 5(a)). At 60 *μ*M (12.25 *μ*g/mL) DHNA, increase in G0/G1 cell population (39.3%) was accompanied by a reduction in G2/M phase cell (12.4%). Increasing DHNA to 120 *μ*M (24.5 *μ*g/mL) increased G0/G1 cell population substantially (51.0%) with a concomitant decrease in both S (39.6%) and G2/M phase cells (9.4%). Moreover, induction of apoptosis was demonstrated by the presence of sub-G0/G1 population (19.7%) (Figure 5(a)).

### 3.6. DHNA Increases DNA Fragmentation

**Figure 6:**
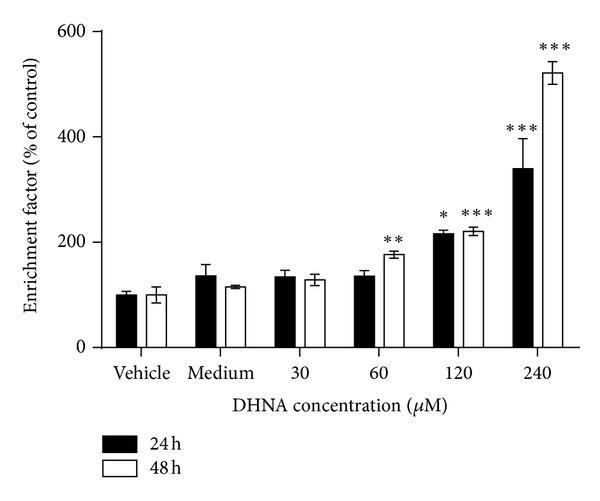
DHNA induces DNA fragmentation in HaCaT cells. HaCaT cells were treated with vehicle (0.24% DMSO), medium only or various concentration of DHNA for 24 and 48 h. Cells were then assayed for DNA fragmentation by the Cell Death Detection ELISA^plus^ Kit. Three independent experiments with triplicates each time were performed with similar results. Data are expressed as mean ± SEM from one representative experiment and significant different at *P < 0.05, **P < 0.01, ***P < 0.001 when versus vehicle control.

After exposure to DHNA, DNA fragmentation in HaCaT cells was analyzed by the Cell Death Detection ELISA^plus^ Kit (Roche Applied Science). As shown in Figure 6, DNA fragmentation was increased in dose-dependent manner. Following treatment of 240 *μ*M (49.01 *μ*g/mL) DHNA, DNA fragmentation in HaCaT cells increased approximately 3.4 and 5.2 folds at 24 and 48 h, respectively, compared with vehicle control.

### 3.7. DHNA Increases TUNEL Positive Cells in HaCaT Cells

**Figure 7:**
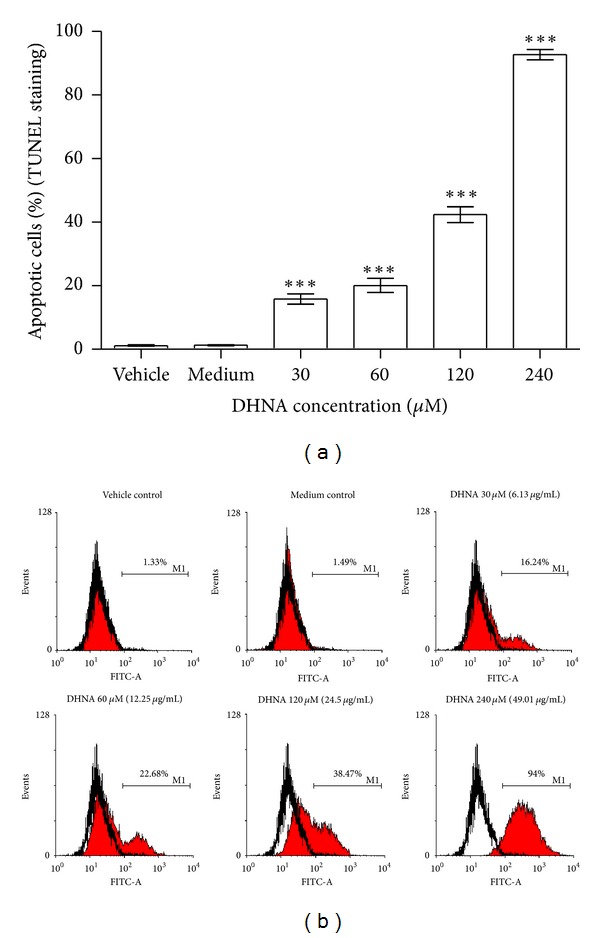
Induction of apoptosis by DHNA in HaCaT cells measured by TUNEL staining. (a) Bar chart and (b) flow cytometric analysis of the effect of DHNA on TUNEL staining in HaCaT cells. HaCaT cells were treated with vehicle (0.24% DMSO), medium only or various concentration of DHNA for 24 h. The cell was analyzed for apoptosis by the *In Situ* Cell Death Detection kit (Fluorescein). In (b), HaCaT cells showing increased fluorescence (TUNEL staining) above that of control population (open graph) are considered apoptotic and their percentage populations are shown. Three independent experiments with triplicates each time were performed with similar results. Data are expressed as mean ± SEM from one representative experiment and significant difference at ***P < 0.001 when versus vehicle control.

In vehicle and medium control-few TUNEL positive (apoptotic) cells presence (1.2 and 1.3%; Figures 7(a) and 7(b)). At all concentration tested (30 to 240 *μ*M/6.13 to 49.01 *μ*g/mL), DHNA dose dependently increased TUNEL positive cells in HaCaT cells from 15.8 to 92.7% (Figures 7(a) and 7(b)). As TUNEL assay is a sensitive method which detects single-strand breaks in high molecular weight DNA, it was able to show that 30 *μ*M (6.13 *μ*g/mL) DHNA treatment induced apoptosis in HaCaT cells as early as 24 h while other apoptotic markers are still not available or detectable (e.g., externalization of PS, morphology).

### 3.8. Western Blot Analysis

**Figure 8:**
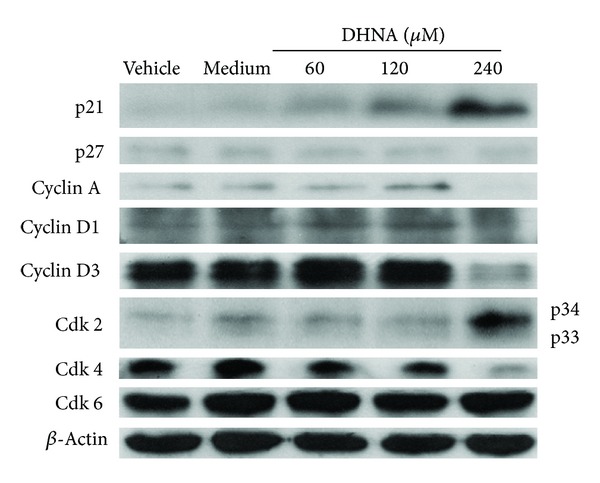
Effects of DHNA on the expression of cell-cycle-related proteins in HaCaT cells. HaCaT cells were treated with vehicle (0.24% DMSO), medium only or various concentrations of DHNA for 12 h and then cells were harvested. Equal amount of cell extracts (10–20 *μ*g) were loaded onto and separated by 12% to 15% SDS-PAGE and analyzed for the expression of cell cycle related proteins by Western blot. Equal protein loading for each sample was monitored by *β*-actin. Data are representative of three reproducible independent experiments.

**Figure 9:**
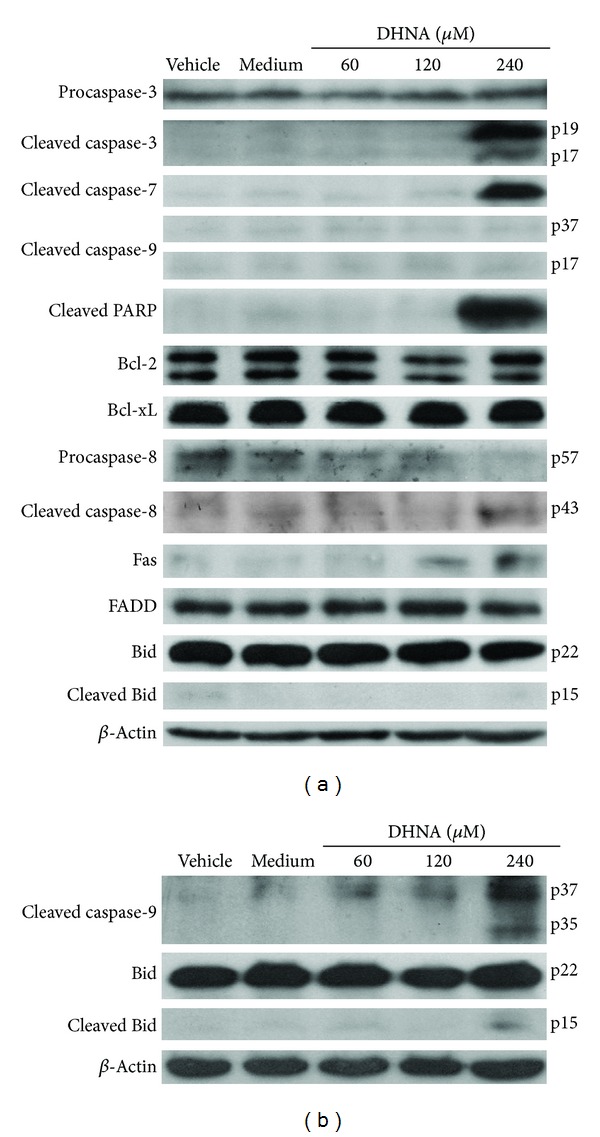
Effects of DHNA on the expression of apoptosis related proteins in HaCaT cells. HaCaT cells were treated with vehicle (0.24% DMSO), medium only or various concentrations of DHNA for (a) 12 and (b) 24 h and then analyzed by Western blot. Equal protein loading was monitored by *β*-actin. Data are representative of three reproducible independent experiments.

In cell cycle related proteins (Figure 8), treatment of HaCaT cells with DHNA for 12 h resulted in a dose-dependent increase in the expression of cyclin dependent kinase inhibitor (Cki) p21. At 240 *μ*M (49.01 *μ*g/mL) DHNA, level of Cyclin A/D1/D3 and Cdk4 which are required for entry into S phase were decreasing. These data further indicated that DHNA arrested HaCaT cells at G0/G1 phase (Figure 5). At the same time, inactive form of Cdk2 (Thr14 and Tyr15 phosphorylated, 34 kDa) was increased while there are no visible change in active form of Cdk2 (Thr160 phosphorylated, 33 kDa). The expression level of p27 and Cdk6 remains unchanged. For apoptosis related proteins (Figure 9), HaCaT cells treated with 240 *μ*M (49.01 *μ*g/mL) DHNA for 12 h decreased procaspase-8 level, upregulated Fas receptor and the active form of caspase-3/7/8 as well as induced PARP cleavage. These findings suggested that DHNA induced HaCaT cells apoptosis through Fas receptor dependent pathway. On the other hands, no active form of caspase-9 was found and Bid was not cleaved. There were also no significant changes in expression levels of Bcl-2, Bcl-xL, Bid, and FADD (Figure 9(a)). When treatment time was increased to 24 h, activated caspase-9 and cleaved Bid could then be detected (Figure 9(b)).

### 3.9. DHNA Induced Caspase Independent Apoptosis in HaCaT Cells

**Figure 10:**
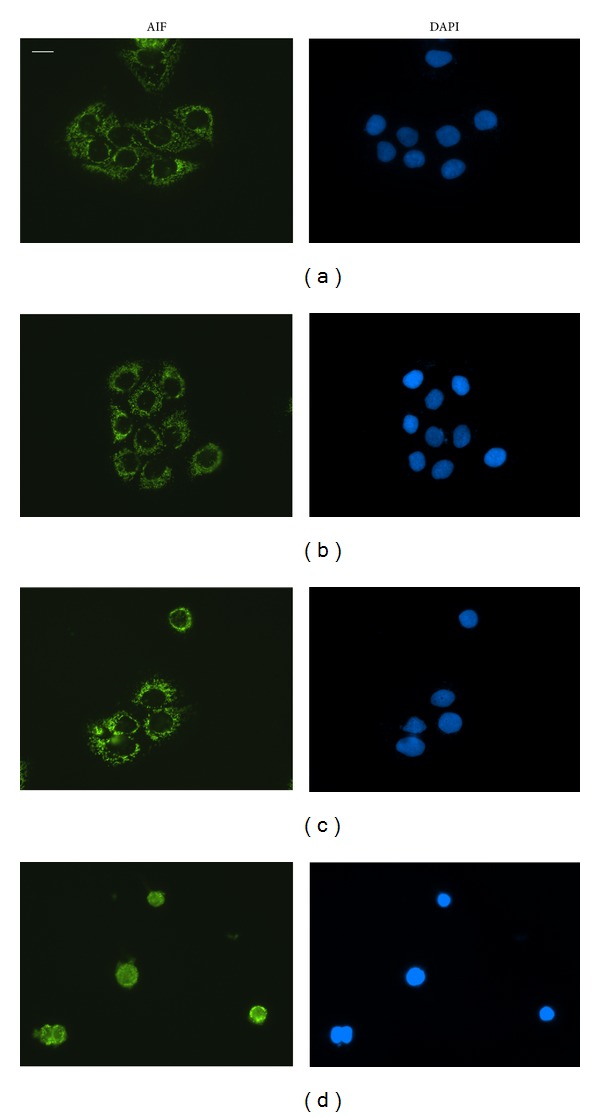
DHNA causes nuclear translocation of AIF in HaCaT cells. HaCaT cells were treated with vehicle (0.24% DMSO; (a)), medium only (b), 120 *μ*M (24.5 *μ*g/mL; (c)) or 240 *μ*M (49.01 *μ*g/mL; (d)) DHNA for 24 h then analyzed by immunofluorescence staining. Cell nuclei were stained by DAPI. Data are representative of three reproducible independent experiments. Scale bar 25 *μ*m.

**Figure 11:**
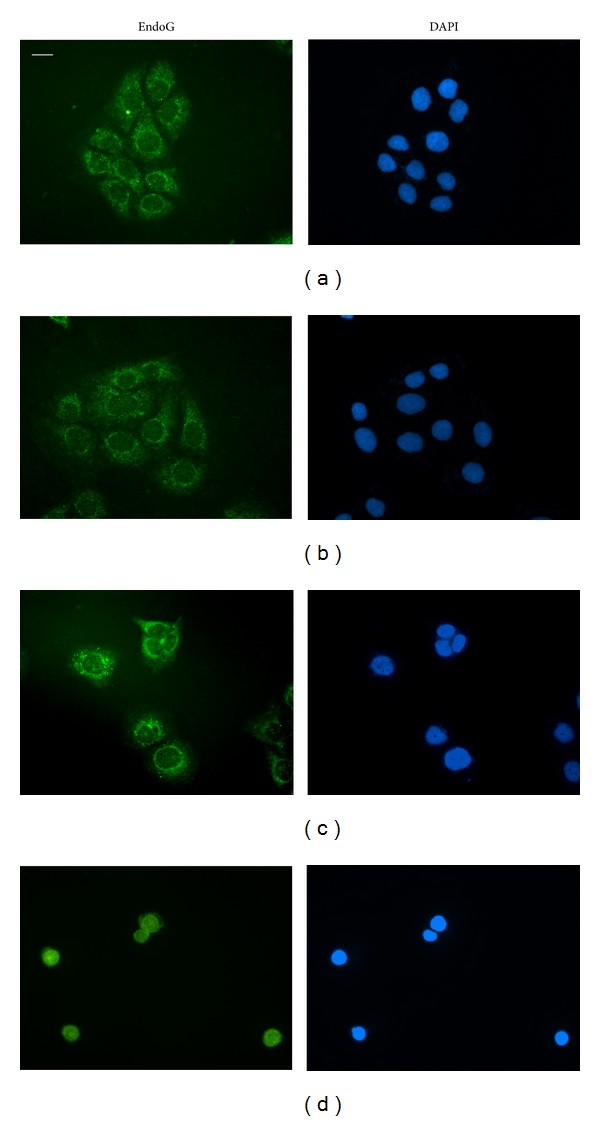
DHNA causes nuclear translocation of endoG in HaCaT cells. HaCaT cells were treated with vehicle (0.24% DMSO; (a)), medium only (b), 120 *μ*M (24.5 *μ*g/mL; (c)) or 240 *μ*M (49.01 *μ*g/mL; (d)) DHNA for 24 h then analyzed by immunofluorescence staining. Cell nuclei were stained by DAPI. Data are representative of three reproducible independent experiments. Scale bar 25 *μ*m.

In vehicle and medium control cells, immunofluorescence staining revealed the localization of AIF in mitochondria and its absence in nucleus (Figures 10(a) and 10(b)), whereas endoG is present in mitochondria as well as in the nucleus (Figures 11(a) and 11(b)). When HaCaT cells were treated with 240 *μ*M (49.01 *μ*g/mL) DHNA for 24 h, both AIF and endoG were translocated to the nucleus (Figures 10(d) and 11(d)). These results suggest DHNA can mediate apoptosis in HaCaT cells in a caspase independent way through the action of AIF and endoG.

### 3.10. IL-1*α* Release Assay

**Figure 12:**
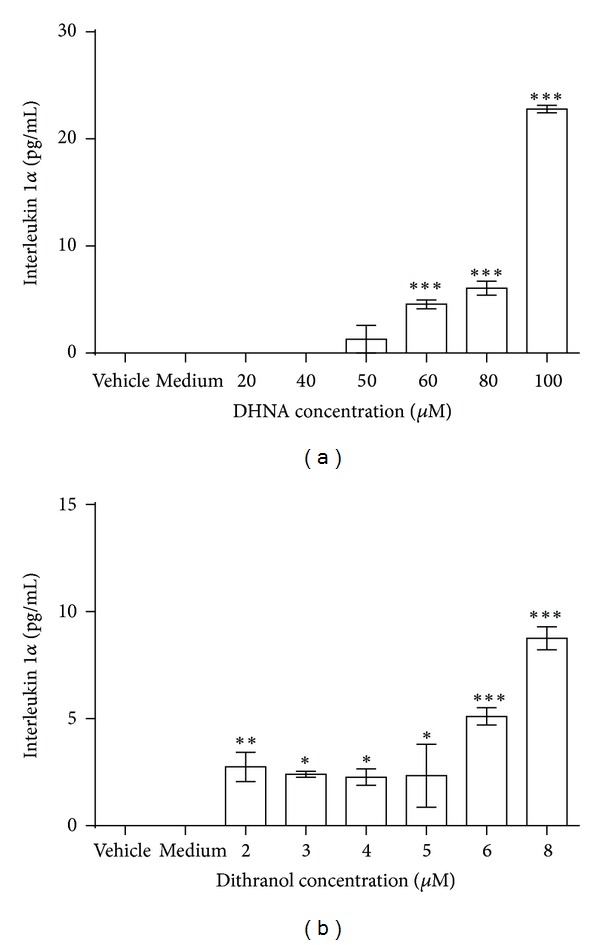
Effect of DHNA and dithranol on IL-1*α* releasefrom NCTC 2544 cells. NCTC 2544 cells were treated with vehicle (0.2% DMSO), medium only or (a) 20 to 100 *μ*M (4.08 to 20.42 *μ*g/mL) DHNA or (b) 2 to 8 *μ*M (0.45 to 1.81 *μ*g/mL) dithranol for 72 h and then assayed for IL-1*α* release by the IL-1*α* ELISA Kit. Three independent experiments with duplicates each time were performed with similar results. Data are expressed as mean ± SEM from one representative experiment and significant difference at *P < 0.05, **P < 0.01, ***P < 0.001 when versus vehicle control.

In vehicle and medium control, no IL-1*α* release from NCTC 2544 cells was detected (Figure 12). DHNA significantly induced the release of this cytokine at a much higher concentration than dithranol (60 *μ*M or 12.25 *μ*g/mL for DHNA versus 2 *μ*M or 0.45 *μ*g/mL for dithranol; ~30 times) (Figure 12). Compared with vehicle control, a significant induction of IL-1*α* release could only be observed at higher concentrations of DHNA tested (60 to 100 *μ*M or 12.25 to 20.42 *μ*g/mL) (Figure 12(a)). In contrast, dithranol induced significant release of this cytokine at all concentrations tested (Figure 12(b)). Hence, at IC_50_ of DHNA on NCTC 2544 keratinocytes (46.80 *μ*M or 9.56 *μ*g/mL; Table 1), no significant IL-1*α* was released, while dithranol readily induced the release of this cytokine at concentrations (2 to 3 *μ*M or 0.45 to 0.68 *μ*g/mL) below its IC_50_ (3.81 *μ*M or 0.86 *μ*g/mL; Table 1).

## 4. Discussion

Psoriasis is recognized as an organ specific autoimmune disease triggered by an activated cellular immune system [22]. As the exact aetiology and pathogenesis remain unknown, it has been argued for many years whether psoriasis is a disorder of the immune system or disorder of the skin. Evidence indicates keratinocytes contributed to the disease [7]. Based on several lines of evidence, we believe that keratinocytes may play a major and important role in the pathogenesis and development of psoriasis. It would be interesting to note that the presence of T cells in skin lesions does not mean it initiates the disease. Although psoriasis is thought to be a Th1 induced disorder, psoriasis develops in patients infected by the human immunodeficiency virus with the same frequency as in the general population [23]. In psoriasis, the expression levels of many gene products are altered, compared with unaffected skin. Among these altered gene products, the expression levels of messenger RNAs transcribed by keratinocytes are much higher than T cells due to the overwhelming number of keratinocytes in lesions [24]. Next, keratinocytes can synthesize many cytokines and chemokines which could induce chemotaxis that attracts and activates leukocytes found in epidermis of psoriatic lesions such as ICAM-1, CD40 and HLA-DR [24, 25]. Increased levels of CXCL9/MIG, CXCL10/IP-10, CXCL11/I-TAC [24], and RANTES/CCL5 [26] synthesized by keratinocytes in psoriasis lesions as well as the BRAK/CXCL14 that are upregulated in nonlesional skin of psoriasis patients [24], may also activate and initiate the migration of mononuclear leukocytes/T cells/monocytes to the psoriatic lesions. Besides, keratinocytes not only produce but also respond to the growth factors and cytokines produced themselves [27]. For example the production of transforming growth factor *α* (TGF*α*) by epidermal keratinocytes is greatly elevated in psoriasis [27]. This results in increased transcription of hyperproliferating keratin K6/16 genes and the activation of keratinocytes, which produce and respond to immunological signals like interleukins 1/6 and growth factors like TGF*α*/*β* and epidermal growth factor (EGF). Subsequently, a series of signal cascades is activated that contributes to the development and persistence of psoriatic lesion. Moreover, the response of psoriatic keratinocytes to T cell cytokine *in vitro* is different from normal keratinocytes. For example, the basal keratinocytes of psoriatic uninvolved skin are susceptible to the growth stimulatory effect of T cell cytokine IFN-*γ*, while this is not in normal skin [28]. Therefore, keratinocytes from a psoriatic environment exist in a preactivated state that is highly responsive to signaling. The special conditions or changes which result in predisposition of activation in those keratinocytes may be the reason for later development to psoriatic plaque under the influence of immunologic stimulation. In this regard, there are genetically engineered mice models that target epidermal keratinocytes and are sufficient to initiate psoriasis like phenotype [29, 30] such as the IKK2 knockout mice [29, 30] and the JunB/c-Jun double knockout mouse [23], and both of them resemble many features of psoriasis that are T cell independent. Taken together, keratinocytes participate in maintaining a chronically perpetuating immune response that sustains psoriasis, and changes in keratinocytes may even trigger this disease in the skin of predisposed individuals. Therefore, it is believed that epidermal alterations may account for the initiation of skin lesions in psoriasis and thus restoring hyperproliferative keratinocytes to normal would be beneficial in this disease. Particularly, inhibition of keratinocytes proliferation and induction of keratinocyte apoptosis have long been considered as targets of antipsoriatic strategies [31], and the discovery of new antiproliferative and keratinocyte differentiation-modulating agents remains a worthy aspect in psoriasis research nowadays.

Earlier we identified the extract of the root of *Rubia cordifolia *L. induces apoptosis in a psoriasis-relevant HaCaT keratinocyte [12, 20, 21]. In particular, hydroxyanthraquinones are the major bioactive compounds in *R. cordifolia *roots [32, 33]. In this study, we attempt to identify the compound which presents in *Rubia cordifolia *L. that may be responsible for the observed effects of the herb extract on HaCaT cells. During screening the anthraquinone precursor, DHNA was found to time and dose dependently inhibit keratinocytes proliferation. Compared with dithranol, DHNA was less potent in inhibiting keratinocytes growth (~4 times on HaCaT; ~12 times on NCTC 2544). However, DHNA exhibited much less cytotoxicity on fibroblasts (~8 times on Hs-68) and melanocytes (~10 times on PIG1) while was comparable to EA extract of *Rubia cordifolia *L. (Table 1). It is worth noting dithranol is safe, effective and has been a mainstay treatment of psoriasis for over 80 years; however, its use has declined because it causes substantial skin irritation as well as staining of skin, clothing, and furniture [34–36]. For topical treatment of psoriasis, skin irritation is a common side effect [1, 4, 35]. Skin irritation is a local arising, reversible, and nonimmunological inflammatory reaction that is caused by contact and stimulation with irritants [37, 38]. Irritants can cause epidermal damage and initiate the release of IL-1*α* from keratinocytes as a primary event in skin defense [39]. Subsequently, secondary mediators (such as release of chemokines) and morphological alterations (vasodilation and infiltration of immune cells) are induced with finally the onset of typical symptoms such as oedema, erythema, itching as well as pain [37, 40]. Despite its substantial skin irritation problem, dithranol is still popular among European countries as an important (second choice) therapy [35, 41]. With respect to the underlying mechanisms of action of dithranol in psoriasis, dithranol can inhibit the growth of mouse and human keratinocytes as well as psoriatic keratinocytes *in vivo*; it was also demonstrated that it induces apoptosis in keratinocytes via the mitochondrial pathway [42]. In this regard, identification of new treatment which can achieve similar apoptotic effects of dithranol while with less irritation side effect would be of great interest.

Traditionally skin irritation assay is conducted in animals using Draize animal test [43] which has been introduced for more than 60 years as a standard for the evaluation of skin irritating properties of chemicals [44]. However for ethical reasons, the Cosmetics Directive (Directive 76/768/EEC) and European Directive 2003/15/EEC impose a permanent ban of animal testing for cosmetic products and ingredients from 2009 in Europe. As the ethical issues and regulatory context are similar around the world [45], hence, there is a great effort aiming to reduce and replace animal studies. The development and use of alternative *in vitro* methods is encouraged [40, 45, 46]. *In vitro* skin irritation studies have been used to predict the skin irritation potential of different chemicals [47, 48]. The most commonly used parameter to assess irritant responses is the* in vitro* measurement of cell viability [37]. It is a suitable endpoint to evaluate the irritation potential of any given substances, as cytotoxicity is known to trigger irritation processes [38]. One *in vitro* skin irritation model, the “single cell assay”, involves a single-cell type cultured in plastic dishes under conventional culture condition [40]. Previous studies suggested that cultured human keratinocytes can be used to predict irritation caused by various surfactants [49] and there is a good correlation between *in vitro* cytotoxicity and *in vivo* skin irritation potential in human [50, 51]. Therefore, single cell assay and cytotoxicity assay have been employed in many studies to predict the skin irritation potential of chemicals. For example, human keratinocyte (HaCaT, NCTC 2544) monolayer cultures have been used as an *in vitro* model to predict potential skin irritation with cytotoxicity as endpoints [39, 52–54]. Therefore in our study, we also used single cell assay with cytotoxicity to evaluate the skin irritation potential of DHNA and dithranol. For better prediction of irritation potential, apart from cell viability, additional biomarkers should also be incorporated in the study. As mentioned, IL-1*α* is an important mediator which initiates inflammation in the skin. Only followed cell injury, IL-1*α* is exclusively released from leaky keratinocytes while in intact epidermis, IL-1*α* is naturally eliminated by desquamation [37]. In both *in vitro* and* in vivo *systems the release of IL-1*α* is accepted as an early marker of irritation [39, 54]. Therefore, together with cell viability test, measuring the distribution of cytokines (release of IL-1*α*) is an effective and promising approach to predict chemicals as potential irritants [39]. The use of keratinocytes with these endpoints as *in vitro* skin irritation model has already been justified [55]. Because of its epidermal location and its ability to synthesize different inflammatory mediators, and there is a good correlation between *in vitro* response of keratinocytes cultures and *in vivo* skin irritation data [53], human keratinocytes become a relevant biological target and, hence, represents an excellent cell model in assessing skin irritants [53]. In EU, the Cosmetics Directive (76/768/EEC) imposes that cosmetic products put on the market within the Community must not cause damage to human health when they are applied under normal of use. Before new cosmetic products are launched and marketed, the potential of its ingredients or the final product in causing skin irritation needs to be evaluated as part of the safety assessment [56]. As mentioned, this directive also bans animal testing on cosmetic products. To replace animal testing the cosmetic and toiletry industry in Europe represented by the European, Cosmetic Toiletry and Perfumery Association (COLIPA), has adopted the use of *in vitro* test systems to predict potential irritants [37]. Since undifferentiated keratinocytes may synthesize higher level of IL-1*α* and its production is decreased as keratinocytes are gradually differentiated [57], therefore the undifferentiated human keratinocytes (NCTC 2544 cells) are used to evaluate the release of this inflammatory cytokine upon treatments with DHNA or dithranol. Our results demonstrated DHNA is predicted to be less irritating than dithranol. First, IC_50_ values from cell viability assay revealed DHNA was less cytotoxic than dithranol (Table 1). On the other hand, both compounds induced the release of proinflammatory cytokine IL-1*α* from keratinocytes; however, DHNA induced the release of this cytokine at a much higher concentration than dithranol (~30 times, Figure 12). Besides, at IC_50_ of DHNA on NCTC 2544 keratinocytes no significant level of IL-1*α* was released (Table 1, Figure 12(a)), while dithranol could readily induce significant release of this cytokine at concentrations below its IC_50_ on NCTC 2544 keratinocytes (Table 1, Figure 12(b)).

Since DHNA has lower skin irritation potential than dithranol, therefore if DHNA can exert similar apoptotic effects on keratinocytes as dithranol, it could be a promising alternative for the treatment of psoriasis. These findings prompt us to investigate whether apoptosis is the underlying mechanism of the antiproliferation effect of DHNA on keratinocytes. Although DHNA is not the single compound which represents the antiproliferation effect of the root extract of *Rubia cordifolia *L. on keratinocytes, this compound may still be one of the many components in the herb extract that are responsible for the observed antiproliferation effects on HaCaT cells and is worthy for further investigations. Since HaCaT keratinocytes represent a model of outer epidermal layers which contact more directly with topically applied psoriasis treatment, subsequent studies were focused on this cell model.

An early event during apoptosis is externalization of PS which is normally confined to the internal leaflet of plasma membrane [58]. This was confirmed in DHNA treated HaCaT cells by annexin V/PI staining (Figures 3(a)–3(d)). A later step in apoptosis is DNA fragmentation that results from the activation of endonucleases [59]. Apoptotic cell shrink, the chromatin becomes pyknotic and compacted, and nucleus breaks up (karyorrhexis). Cell appears to bud (membrane blebbing), which later become apoptotic bodies and phagocytosed by macrophages [60]. Hoechst stained HaCaT cells treated with DHNA clearly revealed chromatin condensation (pyknotic) and nuclear fragmentation (karyorrhexis) which are commonly observed in ongoing apoptotic cells (Figure 2). The Cell Death Detection ELISA^plus^ assay also demonstrated that DHNA treatment increased DNA fragmentation in HaCaT cells by detecting histone-associated DNA fragments (Figure 6). Moreover, induction of apoptosis in HaCaT cells by DHNA was further confirmed with TUNEL technique by detecting DNA strand breaks (Figure 7). It is suggested PS exposure precedes the nuclear changes that define apoptosis as well as the loss of membrane integrity [58]. Our results agreed with this observation that externalization of PS can be detected first (e.g., 60 *μ*M DHNA at 24 h treatment), with the decrease in mitochondrial membrane, potential and morphological change thereafter. Taken together, it is confirmed that the apoptotic induction of HaCaT cells by DHNA is time and dose dependent.

As induction of apoptosis may be cell-cycle related [61, 62], therefore cell-cycle analysis was performed on DHNA treated HaCaT cells. Our investigation showed that DHNA treated HaCaT cells were arrested at G0/G1 phase of the cell cycle with induction of sub-G0/G1 population, which indicates apoptotic cells (Figure 5). Moreover, the resulting G0/G1 arrest was correlated with changes in the expression of major cell cycle proteins as analyzed by theWestern blot. The cyclin dependent kinases (Cdk) are serine/threonine kinases which are important in controlling cell cycle in eukaryotic cells [63–65]. Cyclins form heterodimeric complexes with specific Cdk at different points in cell cycle to phosphorylate target proteins and hereby promote cell cycle progression. In G1/S phase transition, cyclin D/Cdk4 and cyclin D/Cdk6 are the first two complexes to become active and involved in early G1 phase, while cyclin E/Cdk2 is formed in late G1 phase. Later, activated cyclin A/Cdk2 complexes drive cells into S phase. DHNA treatment down-regulated the level of Cyclin A/D1/D3 and Cdk4 which are required for entry into S phase in HaCaT cells (Figure 8). The inactive form of Cdk2 (Thr14 and Tyr15 phosphorylated, 34 kDa) increased while there were no obvious change in the active form of Cdk2 (Thr160 phosphorylated, 33 kDa). Besides, DHNA treatment also dose-dependently increased the expression of cyclin dependent kinase inhibitor p21, which binds and inhibits the activity of cyclin/Cdk complexes and blocks progression through G1/S phase in cell cycle. In psoriasis, epidermal hyperplasia is characterized by an increase of percentage of normally quiescent basal keratinocytes in the proliferative phases of cell cycle [66]. In particular, cyclin D1 is overexpressed in keratinocytes and is associated with epithelial hyperproliferation in psoriasis [67]. Our findings imply DHNA may be able to alter abnormal expression of the cell cycle proteins, favor G0/G1 arrest, and apoptosis in psoriatic keratinocytes which demonstrated decreased apoptosis.

To study the molecular mechanisms underlying DHNA induced HaCaT cells apoptosis, expression level of major caspases and their substrates were examined using the Western blot analysis. DHNA treatment for 12 hours increased the expressions of Fas receptor and the active form of caspase-3/7/8 in HaCaT cells. Besides, the DNA repair enzyme poly (ADP-ribose) polymerase (PARP) was cleaved and inactivated (Figure 9(a)). These results suggest, DHNA induces apoptosis through the death receptor mediated pathway. Ligation of death receptor (e.g., Fas) of the tumor necrosis factor receptor family with their ligands causes rapid death inducing signaling complex (DISC) formation and recruits Fas-associated death domain (FADD), which in turn recruits and processes procaspase-8 into a mature form. Activated caspase-8 then directly activates effector caspase (e.g., caspase 3) to trigger apoptosis [68, 69]. Moreover, mitochondrial permeabilization indicated by the decrease in transmembrane potential was detected in DHNA-treated HaCaT cells (Figure 4). The loss of mitochondrial transmembrane potential is usually associated with cytochrome *c* and other apoptotic factors released from the mitochondria. It is suggested that opening of the mitochondrial permeability transition pore (PTP) complex results in a decrease in MMP and cytochrome *c* release [70]. In mitochondrial apoptotic pathway, permeabilization of mitochondrial outer membrane results in the release of apoptogenic factors (e.g., cytochrome *c*) from intermembrane space into cytoplasm. In cytoplasm, cytochrome *c* forms complex (apoptosome) with apoptotic protease activating factor-1 (Apaf-1) and procaspase-9. The resulting apoptosome activates caspase-9 by cleavage, and activated caspase-9 in turn activates procaspase-3 to mediate apoptosis. The mitochondrial permeabilization is mainly regulated through Bcl-2 family proteins that consist of antiapoptotic (e.g., Bcl-2, Bcl-xL) and proapoptotic members (e.g., Bax). By interacting with PTP complex, the Bcl-2 proteins can regulate mitochondrial permeabilization. In addition, proapoptotic Bcl-2 proteins can also form pores in the outer mitochondrial membrane without involving PTP [70]. Besides, Bcl-2 proteins also provide a link between the death receptor and mitochondrial mediated apoptosis. When cleaved by activated caspase-8, the BH3-only proteins Bid can also trigger cytochrome *c* release by translocation to mitochondria. Since activated caspase-9 and cleaved Bid is only observed after 24-hours-treatment of DHNA (Figure 9(b)), it is postulated that DHNA acts on death receptor first then triggers mitochondrial pathway and, hence, death receptor signaling occurs upstream the mitochondrial events. Further studies are required to confirm our speculation; nevertheless, the role of caspase is confirmed in our model, since the pan-caspase inhibitor Z-VAD-FMK attenuated DHNA-induced HaCaT cells apoptosis (Figure 3(e)).

The results from caspase inhibition assay showed that DHNA induced apoptosis in the HaCaT cells could only be partially blocked with Z-VAD-FMK. Since the caspase inhibitor Z-VAD-FMK used in the experiments is an irreversible inhibitor, therefore caspases alone were not solely responsible for the DHNA induced apoptosis. It would be interesting to note that DHNA treatment results in the loss of mitochondrial transmembrane potential in HaCaT cells (Figure 4). In this regard, the mitochondrial proteins apoptosis inducing factor (AIF) and endonuclease G (endoG) have been implicated in the caspase independent apoptosis [71, 72]; AIF and endoG released from mitochondria can trigger caspase independent apoptotic cell death [73] and contribute to nuclear chromatin condensation and DNA fragmentation [71]. AIF is normally confined to mitochondria. Upon apoptosis induction, AIF translocates from the mitochondrial intermembrane space to the nucleus. In nucleus, AIF causes chromatin condensation and large DNA fragmentation of around 50 kbp [74]. EndoG is mainly present in mitochondrial intermembrane space but it can also be found in nucleus [75]. It is suggested endoG is involved in mitochondrial DNA synthesis as well as apoptosis. Similar to AIF, endoG translocates to the nucleus during apoptosis. When released from mitochondria, endoG cleaves DNA into nucleosomal fragments independent of caspases [76]. Our results provide additional evidences that DHNA also mediates caspase independent apoptosis in HaCaT cells, as AIF and endoG were shown to be translocated to nucleus after DHNA treatment (Figures 10 and 11), where they directly caused chromatin condensation, DNA fragmentation and mediated apoptosis by a caspase independent mechanism [76, 77].

Despite the presently available treatment options, one should bear in mind that psoriasis is a chronic disease and no available therapies are curative in nature. Therefore, disease inevitably recurs when therapy is discontinued [78]. Although most of the therapies in treating psoriasis have significant side effects [79], each medication has its own advantages and limitations. For optimal management, a given treatment requires an initial quick clearing of symptoms followed by long-term maintenance therapy [80]. This involves the sequential use of therapeutic agents which aim at improving the overall outcome of psoriasis treatment as well as to minimize the long-term toxicity of any particular treatment. The strategy includes 3 steps: (i) the clearing stage that employs short-term use of rapidly acting agents; (ii) the transitional stage involves gradual dose decreasing of the rapidly acting agent with the introduction of a safer maintenance agent; and (iii) the maintenance stage with the safer maintenance agent to ensure long-term control with minimal side effects. Our previous and present study suggest that, the ethyl acetate extract of the root of *Rubia cordifolia *L. (EA) and DHNA may be used together in this sequential therapy, in which EA could be effective at producing rapid clearing of psoriatic lesions with the fact that its potency is comparable to dithranol and more potent than DHNA in inhibiting keratinocytes proliferation, whereas DHNA may be better suited for maintenance therapy for its milder irritation effect compared with dithranol.

## 5. Conclusions

As the exact aetiology and pathogenesis of psoriasis is still unknown and current available treatments have many limitations, the pursuit and development of alternative therapeutic options remain an ongoing scientific effort. In this study we identified DHNA, similar to the herb extract we previously identified, induces apoptosis in HaCaT keratinocytes through G0/G1 cell cycle arrest. DHNA also demonstrated to act through both caspase-dependent and caspase-independent pathways of apoptosis. These results indicate DHNA possesses similar apoptotic effects on keratinocytes as dithranol, and at the same time, DHNA shows far less irritating actions as indicated by less cytotoxicity and less potent to induce the release of proinflammatory cytokine IL-1*α*. Since dithranol is an important and popular therapy among European countries, DHNA therefore constitutes a promising alternative agent for treating psoriasis. In addition, our study provides an insight on the potential of using the ethyl acetate extract of the root of *Rubia cordifolia *L. (EA) and DHNA alternatively in a sequential therapy as a safe and effective treatment modality for psoriasis. Future studies on EA and DHNA will further strengthen our knowledge in the mechanisms underlying their antipsoriatic effects and further help the development of drugs for the treatment of psoriasis.

## Figures and Tables

**Table 1:** Comparison of IC_50_ of DHNA, dithranol, and EA on the growth of HaCaT, NCTC 2544, Hs-68, and PIG1 cell lines.

Cell line	Incubation time (h)	IC_50_ in *µ*M (*µ*g/mL)^1^		
		DHNA^2^	Dithranol	EA^3^
HaCaT	48	111.4 (22.75)	24.77 (5.60)	(2.21)
	72	38.94 (7.95)	9.39 (2.12)	(1.49)
NCTC 2544	48	81.37 (16.61)	6.21 (1.41)	(4.51)
	72	46.80 (9.56)	3.81 (0.86)	(2.50)
Hs-68	72	226.3 (46.21)	27.42 (6.20)	(43.95)
PIG1	72	23.98 (4.90)	2.34 (0.53)	(4.80)

^1^At least five different concentrations of compound/extract were used for the calculation of each value. Three independent experiments done in triplicate were performed with similar results.

^2^1,4-dihydroxy-2-naphthoic acid.

^3^Ethyl acetate extract of the root of *Rubia cordifolia *L.

## References

[1] Afifi T, de Gannes G, Huang C, Zhou Y (2005). Topical therapies for psoriasis: evidence-based review. *Canadian Family Physician*.

[2] Griffiths CE, Barker JN (2007). Pathogenesis and clinical features of psoriasis. *The Lancet*.

[3] Traub M, Marshall K (2007). Psoriasis—pathophysiology, conventional, and alternative approaches to treatment. *Alternative Medicine Review*.

[4] Linden KG, Weinstein GD (1999). Psoriasis: current perspectives with an emphasis on treatment. *The American Journal of Medicine*.

[5] Miller DW, Feldman SR (2006). Cost-effectiveness of moderate-to-severe psoriasis treatment. *Expert Opinion on Pharmacotherapy*.

[6] Sizto S, Bansback N, Feldman SR, Willian MK, Anis AH (2009). Economic evaluation of systemic therapies for moderate to severe psoriasis. *The British Journal of Dermatology*.

[7] Yu XJ, Li CY, Dai HY (2007). Expression and localization of the activated mitogen-activated protein kinase in lesional psoriatic skin. *Experimental and Molecular Pathology*.

[8] Kharaeva Z, Gostova E, de Luca C, Raskovic D, Korkina L (2009). Clinical and biochemical effects of coenzyme Q10, vitamin E, and selenium supplementation to psoriasis patients. *Nutrition*.

[9] Heenen M, Simonart T (2008). Apoptosis in psoriatic epidermis. *Journal of Cutaneous Pathology*.

[10] Laporte M, Galand P, Fokan D, De Graef C, Heenen M (2000). Apoptosis in established and healing psoriasis. *Dermatology*.

[11] Boehm I (2006). Apoptosis in physiological and pathological skin: implications for therapy. *Current Molecular Medicine*.

[12] Tse WP, Che CT, Liu K, Lin ZX (2006). Evaluation of the anti-proliferative properties of selected psoriasis-treating Chinese medicines on cultured HaCaT cells. *Journal of Ethnopharmacology*.

[13] Tse TW (2003). Use of common Chinese herbs in the treatment of psoriasis. *Clinical and Experimental Dermatology*.

[14] Koo J, Arain S (1998). Traditional chinese medicine for the treatment of dermatologic disorders. *Archives of Dermatology*.

[15] May BH, Zhang AL, Zhou W (2012). Oral herbal medicines for psoriasis: a review of clinical studies. *The Chinese Journal of Integrative Medicine*.

[16] Li N, Li YQ, Li HY, Guo W, Bai YP (2012). Efficacy of externally applied Chinese herbal drugs in treating psoriasis: a systematic review. *The Chinese Journal of Integrative Medicine*.

[17] Zhang H, Gu J (2007). Progress of experimental study on treatment of psoriasis by Chinese medicinal monomer and single or compound recipe in Chinese Materia Medica. *The Chinese Journal of Integrative Medicine*.

[18] Koo J, Desai R (2003). Traditional Chinese medicine in dermatology. *Dermatologic Therapy*.

[19] Song P, Yan ZF, Xu X (2007). Clinical observation on effect of compound E-bei ointment in treating plaque psoriasis. *Chinese Journal of Integrated Traditional and Western Medicine*.

[20] Tse WP, Cheng CHK, Che CT, Zhao M, Lin ZX (2007). Induction of apoptosis underlies the Radix Rubiae-mediated anti-proliferative action on human epidermal keratinocytes: implications for psoriasis treatment. *International Journal of Molecular Medicine*.

[21] Lin ZX, Jiao BW, Che CT (2010). Ethyl acetate fraction of the root of *Rubia cordifolia* L. inhibits keratinocyte proliferation *in vitro* and promotes keratinocyte differentiation *in vivo*: potential application for psoriasis treatment. *Phytotherapy Research*.

[22] Lowes MA, Bowcock AM, Krueger JG (2007). Pathogenesis and therapy of psoriasis. *Nature*.

[23] Zenz R, Eferl R, Kenner L (2005). Psoriasis-like skin disease and arthritis caused by inducible epidermal deletion of Jun proteins. *Nature*.

[24] Liu Y, Krueger JG, Bowcock AM (2007). Psoriasis: genetic associations and immune system changes. *Genes and Immunity*.

[25] Kanda N, Watanabe S (2006). Suppressive effects of antimycotics on tumor necrosis factor-*α*-induced CCL27, CCL2, and CCL5 production in human keratinocytes. *Biochemical Pharmacology*.

[26] Zhang M, Zhu L, Feng Y, Yang Y, Liu L, Ran Y (2008). Effects of acitretin on proliferative inhibition and RANTES production of HaCaT cells. *Archives of Dermatological Research*.

[27] Jiang CK, Magnaldo T, Ohtsuki M, Freedberg IM, Bernerd F, Blumenberg M (1993). Epidermal growth factor and transforming growth factor *α* specifically induce the activation- and hyperproliferation-associated keratins 6 and 16. *Proceedings of the National Academy of Sciences of the United States of America*.

[28] Chen G, McCormick TS, Hammerberg C, Ryder-Diggs S, Stevens SR, Cooper KD (2001). Basal keratinocytes from uninvolved psoriatic skin exhibit accelerated spreading and focal adhesion kinase responsiveness to fibronectin. *Journal of Investigative Dermatology*.

[29] Danilenko DM (2008). Preclinical models of psoriasis. *Veterinary Pathology*.

[30] Boehncke WH, Schön MP (2007). Animal models of psoriasis. *Clinics in Dermatology*.

[31] Lin J, Liu X, Bao Y, Hou S, An L, Lin X (2008). Effects of isocamptothecin, a novel camptothecin analogue, on proliferation, apoptosis and telomerase activity in HaCaT cells. *Experimental Dermatology*.

[32] Cai Y, Sun M, Xing J, Corke H (2004). Antioxidant phenolic constituents in roots of Rheum officinale and *Rubia cordifolia*: structure-radical scavenginq activity relationships. *Journal of Agricultural and Food Chemistry*.

[33] Singh R, Chauhan SM (2004). 9,10-Anthraquinones and other biologically active compounds from the genus Rubia. *Chemistry & biodiversity*.

[34] Lebwohl M, Ali S (2001). Treatment of psoriasis. Part 1. Topical therapy and phototherapy. *Journal of the American Academy of Dermatology*.

[35] Menter A, Griffiths CE (2007). Current and future management of psoriasis. *The Lancet*.

[36] McBride SR, Walker P, Reynolds NJ (2003). Optimizing the frequency of outpatient short-contact dithranol treatment used in combination with broadband ultraviolet B for psoriasis: a randomized, within-patient controlled trial. *The British Journal of Dermatology*.

[37] Welss T, Basketter DA, Schröder KR (2004). *In vitro* skin irritation: facts and future. State of the art review of mechanisms and models. *Toxicology in Vitro*.

[38] Cotovio J, Grandidier MH, Portes P, Roguet R, Rubinstenn G (2005). The *in vitro* acute skin irritation of chemicals: optimisation of the EPISKIN prediction model within the framework of the ECVAM validation process. *ATLA Alternatives to Laboratory Animals*.

[39] Sanchez L, Mitjans M, Infante MR, Vinardell MP (2006). Determination of interleukin-1*α* in human NCTC 2544 keratinocyte cells as a predictor of skin irritation from lysine-based surfactants. *Toxicology Letters*.

[40] Gibbs S (2009). *In vitro* irritation models and immune reactions. *Skin Pharmacology and Physiology*.

[41] Roelofzen JHJ, Aben KKH, Khawar AJM, Van De Kerkhof PCM, Kiemeney LALM, Van Der Valk PGM (2007). Treatment policy for psoriasis and eczema: a survey among dermatologists in the Netherlands and Belgian Flanders. *The European Journal of Dermatology*.

[42] McGill A, Frank A, Emmett N, Turnbull DM, Birch-Machin MA, Reynolds NJ (2005). The anti-psoriatic drug anthralin accumulates in keratinocyte mitochondria, dissipates mitochondrial membrane potential, and induces apoptosis through a pathway dependent on respiratory competent mitochondria. *FASEB Journal*.

[43] Draize JH, Woodard G, Calvery HO (1944). Methods for the study of irritation and toxicity of substances applied topically to the skin and mucous membranes. *Journal of Pharmacology and Experimental Therapeutics*.

[44] Mizumoto N, Mummert ME, Shalhevet D, Takashima A (2003). Keratinocyte ATP release assay for testing skin-irritating potentials of structurally diverse chemicals. *Journal of Investigative Dermatology*.

[45] Garthoff B (2005). Alternatives to animal experimentation: the regulatory background. *Toxicology and Applied Pharmacology*.

[46] Eun HC, Suh DH (2000). Comprehensive outlook of *in vitro* tests for assessing skin irritancy as alternatives to Draize tests. *Journal of Dermatological Science*.

[47] Kodithala K, Hopfinger AJ, Thompson ED, Robinson MK (2002). Prediction of skin irritation from organic chemicals using membrane-interaction QSAR analysis. *Toxicological Sciences*.

[48] Lee JK, Kim DB, Kim JI, Kim PY (2000). *In vitro* cytotoxicity tests on cultured human skin fibroblasts to predict skin irritation potential of surfactants. *Toxicology in Vitro*.

[49] Korting HC, Herzinger T, Hartinger A, Kerscher M, Angerpointner T, Maibach HI (1994). Discrimination of the irritancy potential of surfactants *in vitro* by two cytotoxicity assays using normal human keratinocytes, HaCaT cells and 3T3 mouse fibroblasts: correlation with *in vivo* data from a soap chamber assay. *Journal of Dermatological Science*.

[50] Osborne R, Perkins MA (1994). An approach for development of alternative test methods based on mechanisms of skin irritation. *Food and Chemical Toxicology*.

[51] Wilhelm KP, Böttjer B, Siegers CP (2001). Quantitative assessment of primary skin irritants *in vitro* in a cytotoxicity model: comparison with *in vivo* human irritation tests. *The British Journal of Dermatology*.

[52] Sanchez L, Mitjans M, Infante MR, Vinardell MP (2006). Potential irritation of lysine derivative surfactants by hemolysis and HaCaT cell viability. *Toxicology Letters*.

[53] Martinez V, Corsini E, Mitjans M, Pinazo A, Vinardell MP (2006). Evaluation of eye and skin irritation of arginine-derivative surfactants using different *in vitro* endpoints as alternatives to the *in vivo* assays. *Toxicology Letters*.

[54] Parodi A, Sanguineti R, Catalano M (2010). A comparative study of leukaemia inhibitory factor and interleukin-1*α* intracellular content in a human keratinocyte cell line after exposure to cosmetic fragrances and sodium dodecyl sulphate. *Toxicology Letters*.

[55] Corsini E, Primavera A, Marinovich M, Galli CL (1998). Selective induction of cell-associated interleukin-1*α* in murine keratinocytes by chemical allergens. *Toxicology*.

[56] Zuang V, Alonso MA, Botham PA (2005). Skin irritation and corrosion. *Alternatives to Laboratory Animals*.

[57] Luo D, Min W, Wu D (2004). IL-1*α* and ATP mRNA expression after ultraviolet irradiation in human keratinocyte original-SCC 12F cells. *The Chinese Medical Journal*.

[58] Martin SJ, Reutelingsperger CPM, McGahon AJ (1995). Early redistribution of plasma membrane phosphatidylserine is a general feature of apoptosis regardless of the initiating stimulus: inhibition by overexpression of Bcl-2 and Abl. *Journal of Experimental Medicine*.

[59] Enari M, Sakahira H, Yokoyama H, Okawa K, Iwamatsu A, Nagata S (1998). A caspase-activated DNase that degrades DNA during apoptosis, and its inhibitor ICAD. *Nature*.

[60] Ihara T, Yamamoto T, Sugamata M, Okumura H, Ueno Y (1998). The process of ultrastructural changes from nuclei to apoptotic body. *Virchows Archiv*.

[61] Adhami VM, Aziz MH, Reagan-Shaw SR, Nihal M, Mukhtar H, Ahmad N (2004). Sanguinarine causes cell cyle blockade and apoptosis of human prostate carcinoma cells via modulation of cylin kinase inhibitor-cyclin-cyclin-dependent kinase machinery. *Molecular Cancer Therapeutics*.

[62] Chow SKY, Chan JYW, Fung KP (2004). Inhibition of cell proliferation and the action mechanisms of arsenic trioxide (As2O3) on human breast cancer cells. *Journal of Cellular Biochemistry*.

[63] Gartel AL, Tyner AL (2002). The role of the cyclin-dependent kinase inhibitor p21 in apoptosis. *Molecular cancer therapeutics*.

[64] Jada SR, Matthews C, Saad MS (2008). Benzylidene derivatives of andrographolide inhibit growth of breast and colon cancer cells *in vitro* by inducing G1 arrest and apoptosis. *The British Journal of Pharmacology*.

[65] Mihara M, Shintani S, Nakashiro KI, Hamakawa H (2003). Flavopiridol, a cyclin dependent kinase (CDK) inhibitor, induces apoptosis by regulating Bcl-x in oral cancer cells. *Oral Oncology*.

[66] Bata-Csorgo Z, Hammerberg C, Voorhees JJ, Cooper KD (1993). Flow cytometric identification of proliferative subpopulations within normal human epidermis and the localization of the primary hyperproliferative population in psoriasis. *Journal of Experimental Medicine*.

[67] Farkas A, Kemeny L, Szell M, Dobozy A, Bata-Csorgo Z (2003). Ethanol and acetone stimulate the proliferation of HaCaT keratinocytes: the possible role of alcohol in exacerbating psoriasis. *Archives of Dermatological Research*.

[68] Igney FH, Krammer PH (2002). Death and anti-death: tumour resistance to apoptosis. *Nature Reviews Cancer*.

[69] Zimmermann KC, Green DR (2001). How cells die: apoptosis pathways. *Journal of Allergy and Clinical Immunology*.

[70] Gukovskaya AS, Pandol SJ (2004). Cell death pathways in pancreatitis and pancreatic cancer. *Pancreatology*.

[71] Saelens X, Festjens N, Vande Walle L, Van Gurp M, Van Loo G, Vandenabeele P (2004). Toxic proteins released from mitochondria in cell death. *Oncogene*.

[72] Vermeulen K, van Bockstaele DR, Berneman ZN (2005). Apoptosis: mechanisms and relevance in cancer. *Annals of Hematology*.

[73] Düssmann H, Kögel D, Rehm M, Prehn JHM (2003). Mitochondrial membrane permeabilization and superoxide production during apoptosis: a single-cell analysis. *Journal of Biological Chemistry*.

[74] Lorenzo HK, Susin SA, Penninger J, Kroemer G (1999). Apoptosis inducing factor (AIF): a phylogenetically old, caspase-independent effector of cell death. *Cell Death and Differentiation*.

[75] Huang KJ, Ku CC, Lehman IR (2006). Endonuclease G: a role for the enzyme in recombination and cellular proliferation. *Proceedings of the National Academy of Sciences of the United States of America*.

[76] Li LY, Luo L, Wang XD (2001). Endonuclease G is an apoptotic DNase when released from mitochondria. *Nature*.

[77] Zhang W, Zhang C, Narayani N, Du C, Balaji KC (2007). Nuclear translocation of apoptosis inducing factor is associated with cisplatin induced apoptosis in LNCaP prostate cancer cells. *Cancer Letters*.

[78] Carey W, Glazer S, Gottlieb AB (2006). Relapse, rebound, and psoriasis adverse events: an advisory group report. *Journal of the American Academy of Dermatology*.

[79] Endzweig-Gribetz CH, Brady C, Lynde C, Sibbald D, Lebwohl M (2002). Drug interactions in psoriasis: the pros and cons of combining topical psoriasis therapies. *Journal of Cutaneous Medicine and Surgery*.

[80] Koo J (1999). Systemic sequential therapy of psoriasis: a new paradigm for improved therapeutic results. *Journal of the American Academy of Dermatology*.

